# Transient Response of Olaparib on Pulmonary Artery Sarcoma Harboring Multiple Homologous Recombinant Repair Gene Alterations

**DOI:** 10.3390/jpm11050357

**Published:** 2021-04-29

**Authors:** Chiao-En Wu, Ca Tung Ng, Kien Thiam Tan

**Affiliations:** 1 Division of Hematology-Oncology, Department of Internal Medicine, Chang Gung Memorial Hospital at Linkou, Chang Gung University College of Medicine, Taoyuan 333, Taiwan; 2 ACT Genomics Co., Ltd., Taipei 114, Taiwan; catungng@actgenomics.com (C.T.N.); jtchen@actgenomics.com (K.T.T.)

**Keywords:** PARP inhibitor, olaparib, next-generation sequencing (NGS), comprehensive genetic profiling (CGP), primary pulmonary artery sarcoma (PPAS), homologous recombination repair (HRR)

## Abstract

Primary pulmonary artery sarcoma (PPAS) is a rare malignancy arising from mesenchymal pulmonary artery cells and mimics pulmonary embolism. Palliative chemotherapy such as anthracycline- or ifosfamide-based regimens and targeted therapy are the only options. However, the evidence of clinically beneficial systemic treatment is scarce. Here, we report a case of disseminated PPAS achieving clinical tumor response to olaparib based on comprehensive genetic profiling (CGP) showing genetic alterations involving DNA repair pathway. This provides supportive evidence that olaparib could be a promising therapeutic agent for patients with disseminated PPAS harboring actionable haploinsufficiency of DNA damage repair (DDR).

## 1. Introduction

Primary pulmonary artery sarcoma (PPAS) is a rare malignancy, with an incidence rate of 1–30 per 100,000 people [1], which is originated from the pulmonary artery’s mesenchymal cells and mimicking pulmonary embolism [1,2,3]. Due to the ambiguous and rare manifestations of clinical image findings, PPAS is frequently misdiagnosed as various pulmonary thromboembolic diseases (e.g., chronic thromboembolic, pulmonary thromboembolism, or chronic thromboembolic pulmonary hypertension) [4]. As such, PPAS has a poor prognosis, with a median overall survival of less than 3 months without surgical resection and 1–2 years in the case with surgical resection [5,6].

Although surgical resection has been demonstrated to prolong survival [2,7], the immediate postoperative mortality rate has been reported to be ~13–15%, and surgical resection nearly never achieves R0 resection [3]. Furthermore, surgery has no role in disseminated PPAS. Unfortunately, palliative therapy (e.g., anthracycline-/ifosfamide-based chemotherapy regimens or targeted therapy) remains limited and unproven [8]. A clinically useful systematic targeted approach is crucial [9].

Herein, we present our experience of treating a patient with PPAS harboring *MDM2* amplification; *CDKN2A* homozygous deletion; and *RAD50, PTCH1, PTEN, CHEK1, MRE11, BRCA2, RB1,* *and BLM* hemizygous deletion, treated with next-generation sequencing (NGS)-guided olaparib (Lynparza^®^) and achieving a transient partial response.

## 2. Case Report

**Figure 1:**
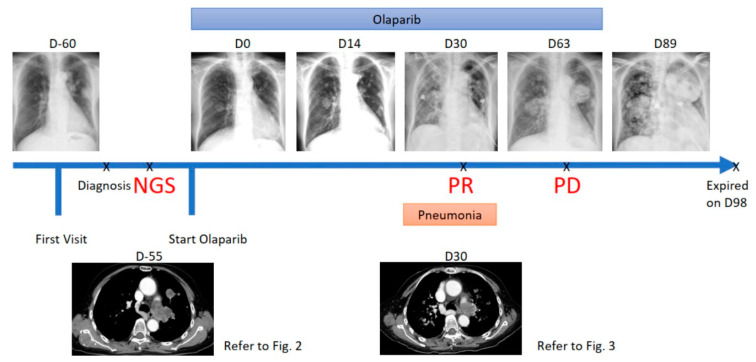
Summary of the clinical course. D0 indicates the first day of olaparib treatment.

**Figure 2:**
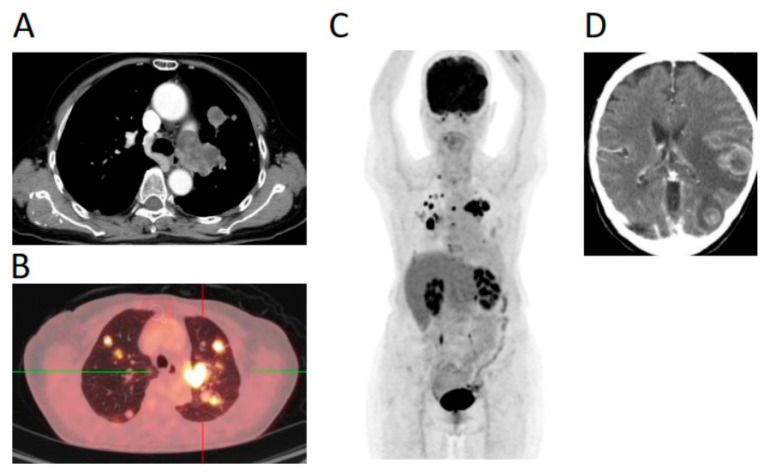
CT images (**A**,**D**) and PET/CT scans (**B**,**C**). (**A**,**B**) Primary pulmonary artery sarcoma (PPAS) with lung metastases by CT scan (**A**) and the corresponding PET/CT scan (**B**). (**C**) A whole-body PET scan showed PPAS with multiple metastases to the lung, bone, and brain. (**D**) Brain metastases on CT scan.

A 71-year-old woman had progressively exertional dyspnea associated with productive cough and chest tightness for half a year. A computed tomography (CT) scan of the chest revealed a left main pulmonary arterial embolism and multiple metastatic nodules over both lung fields (Figure 1 and Figure 2). A CT-guided biopsy was performed for lung masses and pathology reported spindle-cell sarcoma, which was strongly positive for SMA, MDM2, and negative for cytokeratin (AE1/AE3), CAM5.2, CD117 (c-KIT), DOG-1, EMA (E29), P40, S-100, STAT6, TLE1, ER (6F11), PR (1A6), HMB-45, and CDK4. The positivity of SMA indicates muscular differentiation. After reviewing the CT scan and performing positron emission tomography (PET)/CT scan (Figure 2), disseminated PPAS with metastases to both lungs, the left brain, and the T6 vertebra was diagnosed. Palliative whole-brain radiotherapy was subsequently completed and systemic chemotherapy was suggested. However, the patient refused chemotherapy because of the poor performance status of 3 and her old age. As such, NGS-based comprehensive genetic profiling (CGP) was suggested and a lung tumor’s (70% tumor purity) formalin-fixed, paraffin-embedded (FFPE) specimen was sequenced using a targeted panel of 400+ cancer-related genes (ACTOnco^®^+) for molecular-matched therapeutic options [10].

The FFPE lung tumor was subject to next-generation sequencing (NGS) using the ACTOnco^®^ + test (ACT Genomics, Taipei, Taiwan) to detect mutations in the coding region of 440 cancer-related genes and fusion of 31 genes. NGS was conducted at a mean depth of 1298x and 91% uniformity. Twelve nonsynonymous mutations were identified, while none of them was considered clinically significant (Table 1). The germline or somatic origin of these mutations cannot be determined due to lack of a paired normal tissue. No fusion gene was detected.

**Figure 3:**
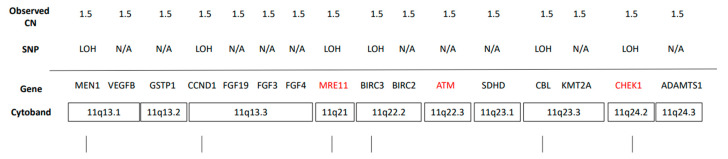
Summary list of genes with single copy loss detected within chromosome 11q.

Clinically relevant copy number variants are shown in Table 2. Amplification of *MDM2*, classified as an oncogene, was identified and confirmed by immunohistochemical (IHC) staining, suggesting that MDM2 is a potential driver in sarcoma. MDM2 inhibitors may be the optimal treatment; however, they are not available outside clinical trials. The homozygous deletion of *CDKN2A* and hemizygous deletion of multiple tumor suppressor genes (TSGs) with haploinsufficiency, including *RAD50* (chr 5)*, PTCH1* (chr 9)*, PTEN* (chr 10)*, CHEK1* (chr 11)*, MRE11* (chr 11)*, BRCA2* (chr 13)*, RB1* (chr 13)*,* and *BLM* (chr 15), were determined by observed copy number < 2 copies and zygosity of SNPs located on the genes (Figure 3). Further analysis showed loss of heterozygosity (LOH) of chromosome 11q, ranging from 11q13.1 to 11q24.3, suggesting single copy loss of another two DNA damage repair (DDR) genes, ATM and H2AX, that localize in this region. Although these DDR genes are all hemizygously deleted, multiple genetic lesions, alterations, and their haploinsufficient nature may lead to homologous recombination deficiency (HRD), as shown by LOH 55.5% of the genomic region interrogated. This indicates that the patient could benefit from a poly(ADP-ribose) polymerase (PARP) inhibitor.

## 3. Treatment Course

**Figure 4:**
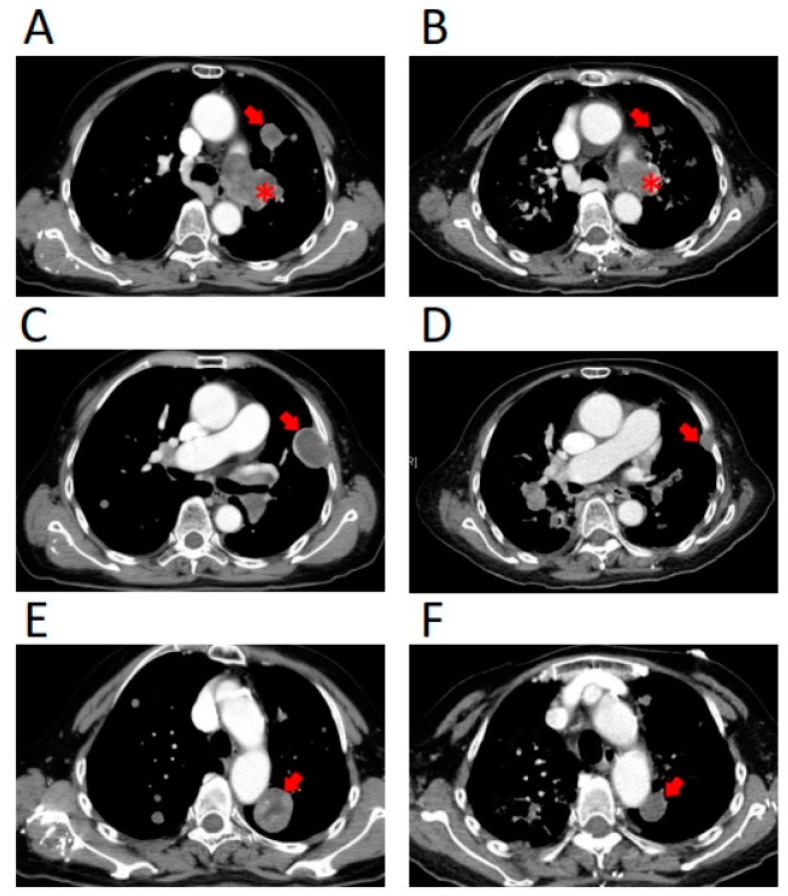
CT scan of the chest revealed a size reduction of tumors before (**A**,**C**,**E**) and after olaparib treatment (**B**,**D**,**F**). The asterisk (*) indicates a primary tumor, while the arrow symbol (→) indicates metastatic tumors. The primary tumor (*) decreased in diameter from 4.8 cm (**A**) to 3.0 cm (**B**) before and after olaparib treatment, respectively. The metastatic tumors of the lung (→) decreased in diameter from 2.0 cm (**A**), 3.4 cm (**C**), and 3.2 cm (**E**) to 0.8 cm (**B**), 1.6 cm (**D**), and 2.3 cm (**F**) before and after olaparib treatment, respectively. Partial response was achieved according to response evaluation criteria in solid tumors (RECIST) criteria.

Based on the genomic alterations identified, olaparib 300 mg BID was prescribed for one week and the dose was reduced to 150 mg BID in the second week due to adverse events of grade 3 anorexia and grade 2 diarrhea. After a two-week treatment, the ECOG performance status improved from 3 to 1 and chest X-ray (CXR) revealed that the lung tumors remained stable (Figure 1). After one month of olaparib treatment, the patient developed a fever and progressive dyspnea, and chest CT revealed infiltrations over both lung fields, but the tumors decreased in size (Figure 1 and Figure 4). The primary and metastatic lung tumors decreased in diameter after olaparib treatment (Figure 4). Partial response was achieved according to the response evaluation criteria in solid tumors (RECIST) criteria. The symptoms of pneumonia improved after the completion of antibiotics. Unfortunately, her symptoms and performance status deteriorated after two months of olaparib treatment, and subsequent CXR confirmed tumor progression. Therefore, olaparib was discontinued, and hospice care was suggested. The patient died three months after the commencement of olaparib treatment.

This report was approved by the Institutional Review Board of Chang Gung Medical Foundation (202100118B0). Written consent was obtained from the patient’s legal guardian to use the images included in this report.

## 4. Discussion

In this case report, a patient with disseminated PPAS was treated with CGP-guided olaparib and achieved a transient partial response. Studies on CGP in PPAS are rare and only reported in abstracts [11,12]. In 21 cases of PPAS, notable alterations that may not be considered actionable included *TP53* (47%), *CDKN2A* (36%), *CDKN2B* (25%), and *RB1* (13%). In contrast, 11% of PPAS harbored clinically relevant genomic alterations that affected *PDGFRA, RICTOR, CDK4,* and *KIT* [11,12]. A total of 10 (48%) of PPAS harboring additional clinically relevant genomic alterations in *EGFR, TSC2, ALK,* and *BRAF* was considered actionable [11,12]. The mean tumor mutation burden (TMB) in the PPAS was 8.3 mutations per Mb (mut/Mb), with 14% of PPAS having TMB > 10 mut/Mb and 10% of PPAS having TMB > 20 mut/Mb, implying that some patients may benefit from immunotherapy [13,14]. No microsatellite instability-high (MSI-H) was found in nine cases with available results. However, no clinical benefit of targeted therapy or immunotherapy was reported based on NGS-based CGP in the literature.

**Figure 5:**
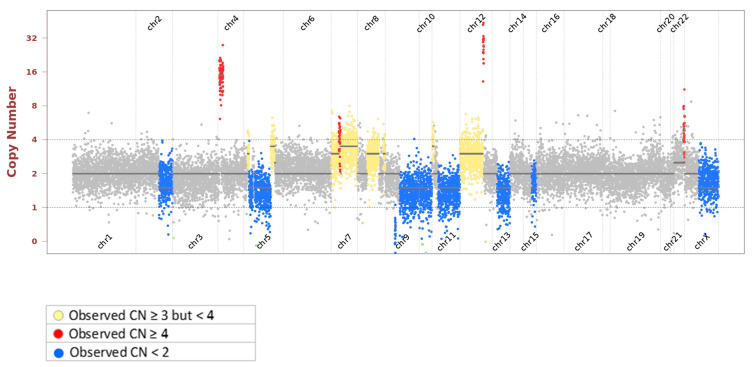
Copy number profile of the lung biopsy sample. The red “dots” indicate amplified regions while the blue “dots” indicate deleterious regions.

In this case, several identified clinically relevant copy number variants provided the rationale for using targeted therapy (Table 2, Figure 5). All of the chromosomal locations and genes for those involved with hemizygous deletions are in Supplementary Table S1. The genomic amplification of the *MDM2* gene in this tumor, as confirmed by IHC, might be one of the primary drivers [15]. *MDM2* amplification was observed in 18.6% of sarcoma cases based on the TCGA database (PanCancer Atlas). Notably, MDM2 amplification is most common in well-differentiated liposarcoma/atypical lipomatous tumors, dedifferentiated liposarcoma, intimal sarcoma, and low-grade osteosarcoma [15].

Moreover, a homozygous deletion of *CDKN2A* was also found, implying that the p14/MDM2/p53 axis played an essential role in tumorigenesis in this particular case. *MDM2* has been reported to be associated with tumorigenesis in multiple solid tumors and sarcoma, as it inhibits the function of p53 (a tumor suppressor) via ubiquitination, leading to proteasomal degradation. Targeting *MDM2* alone or in combination with other agents could be a therapeutic strategy in cancer treatment [16,17]. However, *MDM2* inhibitors are still under investigation in early clinical trials and are clinically unavailable [17,18]. In addition, the homozygous deletion of CDKN2A and hemizygous of RB1 were observed, and CDK4/6 inhibitors (palbociclib, ribociclib, and abemaciclib) may be an off-label option [19]. As a result of the cytostatic effects of CDK4/6 inhibitor monotherapy and insufficient clinical evidence, CDK4/6 inhibitors were not suggested for this patient.

Alternatively, the observed hemizygous deletions of several DDR genes (*RAD50, BRCA2, BLM, CHEK1, MRE11, PTEN, ATM,* and *H2AX)* might highlight a possible therapeutic route. According to Sanmartin et al., haploinsufficiency of DDR genes localized in 11q may confer higher sensitivity to olaparib treatment in neuroblastomas [20]. The collective haploinsufficiency from the 11q-loss would lead to a compromised DDR pathway. As DNA damage can occur at any time or position of the genome, the DDR pathway is essential to safeguard the genome’s integrity [21]. This repair mechanism can be exploited by cancer cells to facilitate the maintenance of cell viability, and a deficiency of this repair mechanism results in cancer susceptibility [22]. This deficiency also indicates the use of PARP inhibitors.

Clinical trials have demonstrated the effective induction of synthetic lethality in tumors harboring *BRCA1*/*2* pathogenic mutations and the loss or disruption of crucial HRR genes [23,24,25]. The copy number loss of BRCAness genes such as *MRE11*, *CHEK1*, *RAD51*, *PTEN* deficiency; knockdown of *RAD50*; and *BLM* have all conferred sensitivity to PARP inhibitors across different tumor types [26,27,28,29,30,31,32]. Furthermore, PARP inhibitors significantly improved survival outcomes in several studies, both as monotherapy (e.g., TOPARP-B and QUADRA) [33,34] and combination therapy (e.g., PAOLA-1/ENGOT-ov25) [35]. These preclinical and clinical studies support expanding different PARP inhibitor treatments to patients with DDR-positive tumor types beyond those with BRCA mutations. Olaparib, an FDA-approved PARP inhibitor, has accumulated evidence establishing a relatively profound and sustained antitumor response across different tumor types, such as ovarian cancer, breast cancer, and pancreatic cancer, associated with germline *BRCA1*/*2* mutations [36]. No documented PPAS cases harboring haploinsufficiency of DDR genes treated with PARP inhibitors have been reported to our knowledge. This case report provides a possible novel strategy for patients with such rare malignancies.

## 5. Conclusions

Given the lack of targeted therapies for patients with PPAS, olaparib could be a promising therapeutic agent for patients with disseminated PPAS harboring actionable haploinsufficiency of DDR genes. Future studies using NGS-based CGP to guide the treatment of PPAS-harboring haploinsufficiency of DDR genes are eagerly awaited.

## Figures and Tables

**Table 1:** Clinically relevant single nucleotide and small indel variants from FFPE lung biopsy.

Gene	Chr	Exon	Accession Number	cDNA Change	Amino Acid Change	Coverage	Allele Frequency	COSMIC ID
*ADAMTS9*	3	1	NM_182920	c.107C > T	P36L	1147	12.7%	-
*ATRX*	X	9	NM_000489	c.1331G > A	R444Q	1626	17.7%	COSM3424965
*BRCA1*	17	10	NM_007294	c.2347A > G	I783V	1301	50.1%	-
*ERBB2*	17	27	NM_004448	c.3763G > T	V1255L	600	47.0%	COSM7313442
*FLT3*	13	7	NM_004119	c.866A > C	N289T	1966	47.2%	-
*KDR*	4	8	NM_002253	c.983C > A	P328H	369	39.6%	COSM3825987
*MUC16*	19	3	NM_024690	c.18096C > G	H6032Q	1438	63.4%	-
*NOTCH4*	6	3	NM_004557	c.316C > A	L106I	1645	49.2%	-
*NSD1*	5	4	NM_022455	c.1070A > G	N357S	1198	47.5%	-
*POLD1*	19	10	NM_001256849	c.1232A > C	Q411P	726	48.5%	-
*RECQL4*	8	-	NM_004260	c.1391-4G > T	Splice region	1495	35.6%	-
*USH2A*	1	66	NM_206933	c.14404T > C	S4802P	1979	54.5%	-

**Table 2:** Clinically relevant copy number variants (CNVs) from archival formalin-fixed, paraffin-embedded lung biopsy.

Pathway Involved	Gene	Copy Number	Alteration
MDM2-P53 pathway	MDM2	39	Amplification
ARF-MDM2-P53 pathway; Rb-E2F-1 pathway; P16/Rb pathway	CDKN2A	0	Homozygous Deletion
Homologous Recombination	RAD50, BRCA2, BLM, CHEK1, MRE11	1	Hemizygous Deletion
Nonhomologous End-joining	RB1	1	Hemizygous Deletion
PTEN-PI3K-AKT pathway	PTEN	1	Hemizygous Deletion
Type I noncanonical Hh pathway	PTCH1	1	Hemizygous Deletion

## Data Availability

The data presented in this study are available on request from the corresponding author. The data are not publicly available due to privacy or ethical issues.

## Supplementary Materials

The following are available online at https://www.mdpi.com/article/10.3390/jpm11050357/s1, Table S1: Chromosomal locations and genes involved associated with hemizygous deletions in Figure 4.
