# Supplementary material for: Transient Response of Olaparib on Pulmonary Artery Sarcoma Harboring Multiple Homologous Recombinant Repair Gene Alterations
Source: J Pers Med. 2021 Apr 29;11(5):357. doi: 10.3390/jpm11050357 (PMC8146095; doi:10.3390/jpm11050357)
Supplement: Supplementary file 1 [file jpm-11-00357-s001.zip › jpm-1195501-supplementary.pdf]

Supplement Table S1. Chromosomal locations and genes involved associated with hemizygous deletions in figure 4

Chromosome	cytoband	gene
chr9	9p21.3	CDKN2A, CDKN2B
chr10	10q11.21	RET
chr10	10q21.2	CDK1
chr10	10q21.3	TET1
chr10	10q23.2	BMPR1A
chr10	10q23.31	FAS
chr10	10q24.2	ABCC2
chr10	10q24.32	SUFU
chr10	10q23.31	PTEN
chr11	11q13.1	MEN1
chr11	11q13.3	CCND1
chr11	11q22.2	BIRC3
chr11	11q23.3	CBL
chr11	11q24.2	CHEK1
chr11	11q21	MRE11
chr13	13q13.1	BRCA2
chr13	13q14.11	TNFSF11
chr13	13q33.1	ERCC5
chr13	13q14.2	RB1
chr15	15q26.1	BLM
chr15	15q26.3	IGF1R
chr2	2q33.1	SF3B1
chr2	2q34	IDH1, ERBB4
chr2	2q36.3	IRS1
chr2	2q37.3	PDCD1
chr5	5p13.1	RICTOR
chr5	5q13.2	CCNB1, CDK7
chr5	5q14.3	CCNH
chr5	5q22.2	APC
chr5	5q31.1	HSPA4, RAD50
chr5	5q31.2	CTNNA1
chr9	9q22.32	PTCH1

chr9	9q21.33	NTRK2
chr9	9q22.2	SYK
chr9	9q22.32	FANCC
chr9	9q33.3	HSPA5
chr9	9q34.12	ABL1
chr9	9q34.2	ADAMTS13, RXRA
chr9	9q34.3	NOTCH1
chrX	Xq21.1	ATRX
